# Numerical Simulation of Gas Phase Reaction for Epitaxial Chemical Vapor Deposition of Silicon Carbide by Methyltrichlorosilane in Horizontal Hot-Wall Reactor

**DOI:** 10.3390/ma14247532

**Published:** 2021-12-08

**Authors:** Botao Song, Bing Gao, Pengfei Han, Yue Yu, Xia Tang

**Affiliations:** The Institute of Technological Sciences, Wuhan University, Wuhan 430072, China; 2020106520027@whu.edu.cn (B.S.); 2020106520028@whu.edu.cn (P.H.); 2019106520025@whu.edu.cn (Y.Y.); 2018106520022@whu.edu.cn (X.T.)

**Keywords:** chemical vapor deposition, gas phase reaction, MTS/H_2_ ratio, numerical model

## Abstract

Methyltrichlorosilane (CH_3_SiCl_3_, MTS) has good performance in stoichiometric silicon carbide (SiC) deposition and can be facilitated at relatively lower temperature. Simulations of the chemical vapor deposition in the two-dimensional horizontal hot-wall reactor for epitaxial processes of SiC, which were prepared from MTS-H_2_ gaseous system, were performed in this work by using the finite element method. The chemistry kinetic model of gas-phase reactions employed in this work was proposed by other researchers. The total gas flow rate, temperature, and ratio of MTS/H_2_ were the main process parameters in this work, and their effects on consumption rate of MTS, molar fraction of intermediate species and C/Si ratio inside the hot reaction chamber were analyzed in detail. The phenomena of our simulations are interesting. Both low total gas flow rate and high substrate temperature have obvious effectiveness on increasing the consumption rate of MTS. For all cases, the highest three C contained intermediates are CH_4_, C_2_H_4_ and C_2_H_2_, respectively, while the highest three Si/Cl contained intermediates are SiCl_2_, SiCl_4_ and HCl, respectively. Furthermore, low total gas flow results in a uniform C/Si ratio at different temperatures, and reducing the ratio of MTS/H_2_ is an interesting way to raise the C/Si ratio in the reactor.

## 1. Introduction

Since it has good performance on resistance to high temperature [1] and the ability to work under extremely harsh conditions with exceptional functionalities [2], silicon carbide (SiC) became an attractive material for power electronics and optoelectronics applications [1,3]. However, the deposition quality of SiC is still limited via chemical vapor deposition (CVD) processes [3], which constitute an important technology for the semi-conductor industry [4,5]. Horizontal [5,6,7,8] and vertical [9,10,11,12] reactors were widely used for CVD processes. High temperature CVD process in the hot-wall CVD reactor can achieve relatively high growth rates of SiC [13,14,15]. However, extreme high temperature conditions contributed undesirable increase of defects in the grown SiC films [16]. In this work the gas phase species were reacted in the temperature range of 900–1500 °C. Similar temperature distributions were employed in comparable investigations [12,16,17,18].

Different precursors [18,19,20,21] have been employed for the CVD processes of SiC. In comparison to the traditional CVD gas systems (such as SiH_4_-C_3_H_8_-H_2_ gaseous system), the addition of chloride contained species as precursors leads to an elevated deposition rate and optimized surface morphology [20]. The employment of chloride contained precursors is an elegant way to circumvent the problem with the silicon droplets in the CVD processes [22]. Methyltrichlorosilane (CH_3_SiCl_3_, MTS) is one of the chlorides contained species and can be used as the precursor gas for hot-wall chemical vapor deposition of SiC [17,18,23]. MTS becomes an affordable precursor for CVD processes of SiC since it has good performance in stoichiometric SiC deposition and can be facilitated at relatively lower temperature [12]. It decomposes in carrier gas to intermediate species, of which contribute greatly to deposition of the SiC film on the substrate surface [12,17].

Since researchers are interested in understanding and predicting the overall growth phenomena in CVD processes, calculations [7,12,17,18,20,24] of chemical kinetics coupling with heat and mass transfer and fluid dynamics were commonly used to investigate SiC growth process. Simulation models [12,17,18,23,25] with gas phase reactions of MTS-H_2_ gaseous system were proposed by different researchers. However, thorough investigations focus on the effect of reactor temperature, MTS/H_2_ ratio and total gas flow rate are still lacked. The desired composition of gas phase species inside the reactor greatly affected by the MTS/H_2_ ratio, temperature and total flow rate [26]. Therefore, it is valuable to conduct thorough investigations on these conditions.

In the present work, the CVD model for the epitaxial growth process of SiC in a horizontal hot-wall reactor was simulated by using finite element method, which was widely employed in calculations for thermodynamic, computational fluid dynamics (CFD), and chemistry kinetic.

The carrier gas is hydrogen (H_2_) and the precursor gas is MTS. Different conditions of temperature, MTS/H_2_ ratio and total flow rate were employed in this work, and their effects on the consumption rate of precursor, molar fractions of major intermediates, and C/Si ratios based on important intermediates were analyzed in detail.

## 2. Simulation Modeling

Both chemical reaction kinetics and physical transfer phenomena are contained in these simulations. Additionally, assumptions proposed as [12,17,19]: (1) the mixture flow in the reactor is ideal laminar flow; (2) the gas-phase reactions are very fast; and (3) reaction expansion or contraction was neglected. Normally, physics applied in a CVD process which, using the dilute mixture approach, include three important parts: heat and mass transfer, fluid flow and chemistry [7]. The equations for mass and heat transfer are as follows [12] with time cumulative terms:

(1) $$\rho C_{p}\frac{\partial T}{\partial t}+\nabla \cdot \left(-k\nabla T\right)+\rho C_{p}\vec{u}\cdot \nabla T=Q_{r}$$

(2) $$\frac{\partial \rho }{\partial t}+\nabla \cdot \rho \vec{u}=0$$

(3) $$\rho \frac{\partial \vec{u}}{\partial t}+\rho \vec{u}\cdot \nabla \vec{u}=-\nabla p+\nabla \cdot \left[\mu \nabla \vec{u}+{\left(\nabla \vec{u}\right)}^{T}-\frac{2}{3}\mu \nabla \cdot \vec{u}\vec{I}\right]$$

(4) $$\rho \frac{\partial w_{i}}{\partial t}+\nabla \cdot \left(-\rho D_{i}\nabla cw_{i}-D_{i}^{T}\nabla \left(lnT\right)\right)+\rho \left(\vec{u}\cdot \nabla \right)w_{i}=M_{i}R_{i}$$

Here, Term $\vec{I}$ is the identity tensor, T is temperature, $R_{i}$ is reaction rate, $M_{i}$ is molecular weight, $Q_{r}$ is the external heat sources including radiation, $C_{p}$ is heat capacity, and k is thermal conductivity, and $D_{i}$ and $D_{i}^{T}$ are the gas diffusion coefficient and the thermal diffusion coefficient, respectively. Subscript i denotes the ith gas species. The term $\vec{u}$ is the vector of mass velocity, p is the pressure, ρ is the mixture mass density and here it is assumed as [1] $\rho =\frac{pM}{RT}$, where, R is the ideal gas constant, and M is the molar mass of the mixture gas [19].

The gas diffusivity at various temperatures and pressures was obtained using the kinetic theory of gases, and details can be found in references [26,27,28,29]. The properties of flow and heat transfer in the gas phase proposed by other researchers [7] were employed in this work.

Suppose there are i reactants and j products for each reversible reaction, then the reaction rate $R_{k}$ for a reversible reaction can be given by [4]:

(5) $$Rk=kf\Pi {\left(\frac{Pfi}{RT}\right)}^{vif}-kb\Pi {\left(\frac{Pfj}{RT}\right)}^{vjb}$$

where $f_{i}$ ($f_{j}$) is mole fraction for the ith (jth) reactants (products) in the reaction, the $k_{f}$ and $k_{b}$ are forward reaction rate constant and reverse reaction rate constant, respectively, and $v_{if}$ and $v_{jb}$ are the forward and reverse stoichiometric coefficient, respectively. The reaction rate for each reaction follows the Arrhenius law [19]

(6) $$k_{f,b}=AT^{\beta }\exp\left(\frac{-E}{RT}\right)\;\;\;\;$$

The value of A, β and E can be found in references [12,17]. The reverse reaction rate constant without terms A, β and E can be calculated by [4]:

(7) $$k_{b}=\frac{k_{f}}{k_{eq}}{\left(\frac{RT}{P^{0}}\right)}^{\left(\underset{j}{\sum }v_{jb}-\underset{i}{\sum }v_{if}\right)}\;$$

(8) $$k_{eq}=\exp\left(-\frac{∆H-T∆S}{RT}\right)\;\;$$

where ∆H and ∆S are reaction enthalpy and reaction entropy, respectively. More details of the calculation method of these terms in Equations (5)–(8) can be found in references [4,19].

**Figure 1:**
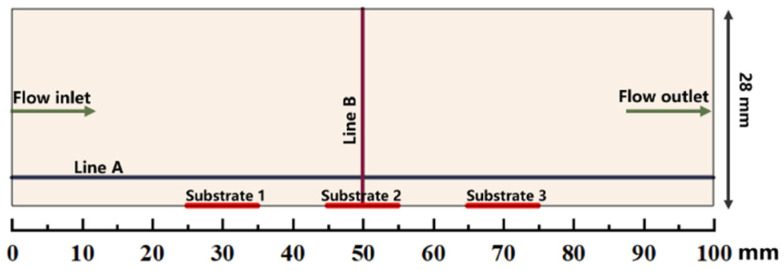
Schematic illustration of the computational gas phase domain above the susceptor.

The simulations are performed in a simplified 2D model of a horizontal hot-wall CVD reactor (28 mm in internal diameter and the susceptor length is 100 mm). The distance from the inlet of quartz tube to susceptor is approximately 450 mm. Only the gas phase region is regarded as a numerical domain in this work [21,30]. The schematic of the computational gas phase domain above the susceptor is illustrated in Figure 1. There are 3 substrates (10 mm × 10 mm) located on the susceptor. The location of substrate 1 is close to flow inlet along the susceptor, while the location of substrate 3 is close to flow outlet along the susceptor. Horizontal line A is about 2 mm distance above the susceptor, and the vertical line B is from the center of substrate 2 to the upper wall. The line A and line B at succedent context imply the line A and line B indicated in Figure 1.

Boundary conditions employed in the simulation model could keep as fixed values [18]. According to the research conditions, a constant temperature of reactor wall was fixed as 900 °C, and temperatures of substrate surfaces were fixed in constant values as listed in Table 1, in which includes the main research conditions in this work. The ratio of MTS/H_2_ at the inlet of quartz tube is 25% for case 1, case 2, case 3 and case 4, and the total gas flow rate is lower for case 1 and case 2 while higher for case 3 and case 4. The ratio of MTS/H_2_ at the inlet of quartz tube is 1.5% for case 5 and case 6. It should be noted that the inlet gas flow rate and the ratio of MTS/H_2_ mentioned in succedent context indicate the gas flow rate and ratio at the inlet of quartz tube. Please note that we focus on the gas phase reactions mechanism in our simulations, the temperature conditions employed in this work are appropriate despite the temperature difference employed in this work either between different substrates or between the substrates and the upper wall may seems exaggerated when compared with experimental situations.

In order to facilitate the generation of regular meshes to improve the convergence of calculation, it is assumed that the substrate is adhered to the susceptor and the effect of geometry is neglected in this model. Since in the present work we focus on the gas phase reactions in the region above the susceptor, these assumptions can be considered reasonable.

The calculation results of gas phase reactions in the research of Kang Guan et al. [12] shows a good agreement with experiments, so it is reasonable to employ these gas phase reactions listed in their work in our simulations. Details of gas phase reaction equations and chemistry kinetics can be found in references [12,17] and not be repeated here. We focus on the gas phase reactions in this work and the effect of surface reaction mechanism is neglected.

## 3. Results and Discussion

As mentioned in Section 2, since we focus on the gas phase reactions in the reactor, the main growth conditions were kept as fixed values in our calculations. Chemistry kinetics, computational fluid dynamics, and heat and mass transfer coupled in the models deliver great contributions in these simulations to calculate the temperature distribution, gas phase velocity distribution, and molar fractions of intermediate species formed inside the reactor for each case. Some of the gas phase intermediates play important role in surface reactions, the C/Si ratio based on these species will also be discussed.

**Figure 2:**
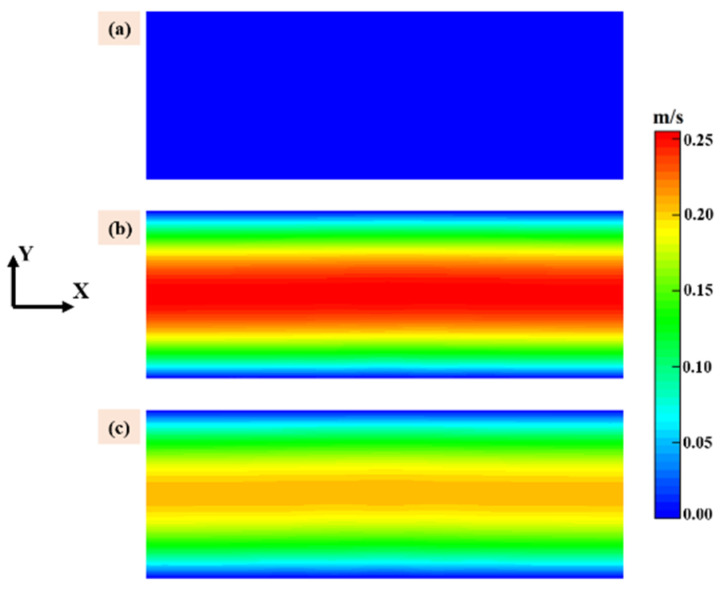
Velocity of the gas phase above the susceptor: (**a**) case 1 and case 2; (**b**) case 3 and case 4; and (**c**) case 5 and case 6.

Velocity of the gas phase above the susceptor is show in Figure 2 with same scale, the X direction is the flow direction, and the color bar for all subfigures is illustrated in the right end. It should be noted that the velocity distribution employed in this work was calculated under the standard conditions based on the total gas flow rate at the inlet of the quartz tube. It is obviously that when the ratio of MTS/H_2_ is fixed at 25% the flow velocity is highest in case 3 and case 4 and lowest in case 1 and case 2 cause the former have the higher carrier gas flow rate. The flow velocity plays an important role in precursor consumption rate and this will be discussed in the following context.

**Figure 3:**
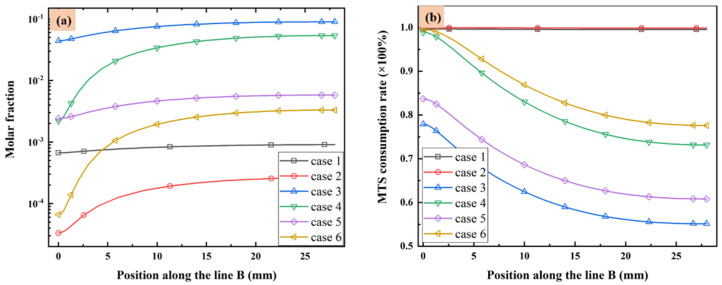
(**a**) MTS molar fraction and (**b**) MTS consumption rate along line B.

The molar fractions of MTS in different cases from substrate to the upper wall along line B are illustrated in Figure 3a. Additionally, the MTS consumption rate defined as the ratio of consumed MTS to the initial MTS fraction is illustrated along line B in Figure 3b. When the MTS/H_2_ ratio is fixed at 25%, the MTS molar fraction increases with the decrease of substrate temperature or increase of total flow rate, since the lower temperature or higher gas flow velocity will reduce MTS consumption rate. When the carrier gas flow rate is fixed at 5000 sccm, reduce the MTS/H_2_ ratio led to more consumption of MTS.

Normally in CVD process, the deposition on substrate surface is usually contributed by intermediates of gas phase reactions instead of precursors [23,25]. It seems when the substrate temperature is high and the total gas flow rate is low, the consumption rate of MTS will increase greatly. Therefore, most of MTS gas will decompose into intermediate species. Decreasing the substrate temperature or increasing either the gas flow rate or the ratio of MTS/H_2_ will reduce the consumption rate of MTS. In the situation that the total gas flow rate is very low (case 1 and case 2), the consumption rate of MTS is close to 1.

**Figure 4:**
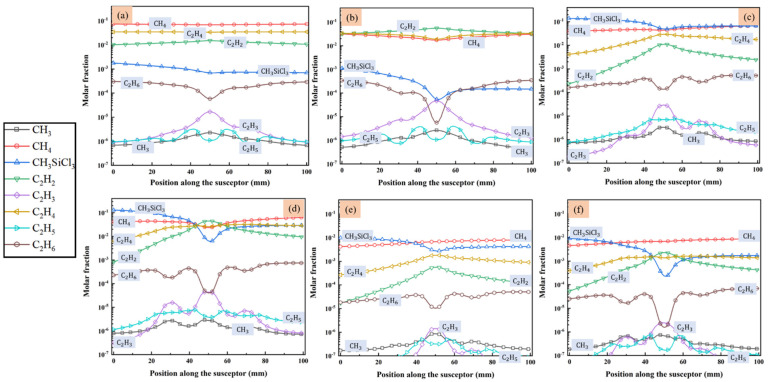
The molar fraction of main species that contained C in the gas phase above the susceptor: (**a**) case 1; (**b**) case 2; (**c**) case 3; (**d**) case 4; (**e**) case 5; and (**f**) case 6.

**Figure 5:**
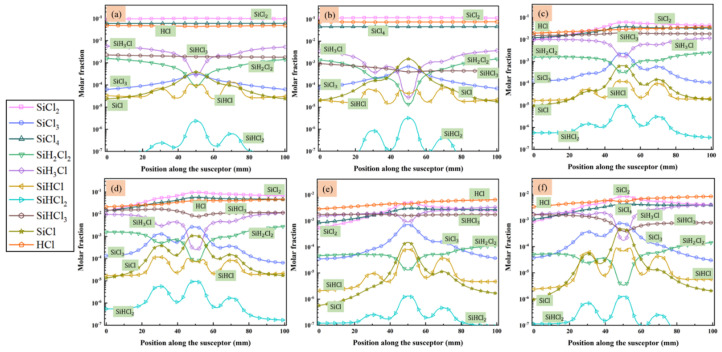
The molar fraction of main species that contained Si/Cl in the gas phase above the susceptor: (**a**) case 1; (**b**) case 2; (**c**) case 3; (**d**) case 4; (**e**) case 5; and (**f**) case 6.

MTS decomposes quickly in the reactor and intermediates are produced. Since we are interested in the compositions of gas phase species close to the substrate surface, the molar fractions of main intermediate species formed above the susceptor are discussed in detail, along line A (as indicated in Figure 1) from gas inlet to outlet. Figure 4 and Figure 5 show the predicted molar fractions of important C contained species and Si/Cl contained species at different cases, respectively. The molar fractions which greater than 10^−7^ are taken along line A from the flow inlet direction. The most three C contained intermediates (major C contained intermediates) are CH_4_, C_2_H_4_ and C_2_H_2_, respectively, while the most three Si/Cl contained intermediates (major Si/Cl contained intermediates) are SiCl_2_, SiCl_4_ and HCl, respectively. It seems that the ratio of MTS/H_2_ and total gas flow rate have significant effect on the fractions for intermediate compositions. The molar fractions of major C contained intermediates are relatively lower than major Si/Cl contained intermediates. The increase of temperature will promote the composing of some intermediate species like C_2_H_2_, SiCl_2_, etc., so the formation of other intermediate species like C_2_H_3_, SiH_3_Cl, etc., will be inhibited.

Besides, when the ratio of MTS/H_2_ is 25%, the molar fractions of major C contained intermediates and major Si/Cl contained intermediates decrease with increase of total flow rate. It is caused by the decrease of MTS consumption rate shown in Figure 3. When the ratio of MTS/H_2_ is 25% at lower gas flow rate, the most abundant C contained intermediate is CH_4_ at lower temperature and changes to C_2_H_2_ with increasing temperature. Furthermore, CH_4_ is the most abundant C contained intermediate for all cases when the carrier gas flow rate is 5000 sccm.

When the ratio of MTS/H_2_ is 25%, the most abundant Si/Cl contained intermediate is SiCl_2_. SiCl_2_ allows higher deposition temperatures and restrains the formation of silicon clusters on the film surface and has high surface activity [20]. Therefore, there is high possibility of chemically stability in cases 1–4. HCl becomes the most abundant Si/Cl contained intermediate when the ratio of MTS/H_2_ decrease to 1.5%. Researchers [12,31,32] pointed out that HCl highly affects the growth rate and is involved in the deposition because of its etching effect on SiC film. It implies that HCl has a greater effect on the growth rate at low MTS/H_2_ ratio in case 5 and case 6 than at high MTS/H_2_ ratio in case 3 and case 4.

Researchers [12,17] have proposed a serious of modified surface reactions based on the MTS-H_2_ gaseous system. Surface reactions in which C or Si contained intermediate species participate as reactants are listed in Table 2, including atom adsorption reactions, and reactions between gaseous and surface species.

It can be seen, C contained gas phase intermediates consume empty sites on Si surfaces then produce surface species with C sites (C(S) and CH(S)). Additionally, Si contained gas phase intermediates consume empty sites on both C and Si surfaces then produce surface species with Si sites (SiCl(S), Si(S), H(S) and Cl_C_(S)) and C sites (Cl_Si_(S)). Therefore, these gas phase intermediates listed in Table 3 participate as reactants in surface reactions contribute directly to produce surface species.

**Figure 6:**
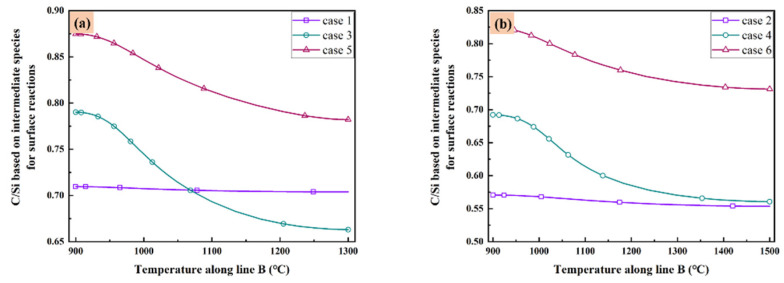
C/Si ratio based on intermediate species for surface reactions as the function of temperature along line B: (**a**) case 1, case 3 and case 5; and (**b**) case 2, case 4 and case 6.

As listed in Table 3, there are five C contained species and eight Si contained species play important role in the surface reaction model. These species are the most important intermediate species contributing to the surface reactions in gas phase. Therefore, it is valuable to discuss the distribution of C/Si ratios based on all above species in the reactor of all cases. The C/Si ratios for all cases based on all the intermediate species in Table 3 are illustrated in Figure 6 as the function of temperature along line B. It seems the low temperature corresponding to the high C/Si ratio in all cases. When the total gas flow rate is low enough as in case 1 and case 2, the difference of C/Si ratio is very small at different temperature. Additionally, when the total gas flow rate is fixed, higher substrate temperature means lower C/Si ratio. However, if you have a preference of higher C/Si ratio, increasing the total gas flow rate may satisfy your purpose when the substrate temperature is high as 1500 °C. Furthermore, decrease the MTS/H_2_ ratio within a reasonable range seems a more elegance way since both case 5 and case 6 have higher C/Si ratio at different substrate temperatures.

## 4. Conclusions

Simulations of the MTS-H_2_ gaseous system occurring in a two-dimensional horizontal hot-wall CVD reactor model were calculated by using finite element method. The gas phase reaction equations and kinetics were obtained from the literature, and surface reaction kinetics were not considered. The temperature range of substrates was 1000–1500 °C, the range of carrier gas flow rates was 100–5000 sccm, and the ratio of MTS/H_2_ was fixed at 25% or 1.5%.

The results of simulations indicated that both the temperature and total flow rate show a distinct effect on consumption rate of MTS. For all cases, CH_4_, C_2_H_4_, C_2_H_2_, SiCl_2_ and SiCl_4_ are the abundant C or Si contained intermediate species in the reactor. Increasing temperature will promote the composing of some intermediates like C_2_H_2_ and SiCl_2_, so the formation of other intermediates like C_2_H_3_ and SiH_3_Cl will be inhibited. HCl which was proved highly affecting the growth rate in other literatures becomes to be the most abundant Si/Cl contained intermediate when the ratio of MTS/H_2_ is 1.5%. The C/Si ratio based on intermediates which participate as reactants in surface reactions in the reactor, becomes uniform at different temperatures when the total gas flow rate is low, and increases when reducing the ratio of MTS/H_2_.

## Figures and Tables

**Table 1:** Calculation conditions in this work.

	Substrate 1 Temp (°C)	Substrate 2 Temp (°C)	Substrate 3 Temp (°C)	H_2_ Flow (sccm)	MTS Flow (sccm)	Pressure (kPa)
Case 1	1000	1300	1100	100	25	6
Case 2	1200	1500	1100	100	25	6
Case 3	1000	1300	1100	5000	1250	20
Case 4	1200	1500	1100	5000	1250	20
Case 5	1000	1300	1100	5000	75	20
Case 6	1200	1500	1100	5000	75	20

**Table 2:** List of surface reactions in which C or Si contained intermediate species participate as reactants from references [12,17].

Reaction on Bulk Silicon Carbide Surface *	
SiHCl_3_ + 2Si($) + 2C($) → SiCl(S) + H(S) + 2Cl_Si_(S)	SiCl_4_ + 2Si($) + 2C($) → SiCl(S) + Cl_C_(S) + 2Cl_Si_(S)
SiHCl_3_ + Si($) + 3C($) → SiCl(S) + H(S) + Cl_C_(S) + Cl_Si_(S)	SiH_2_Cl_2_ + Si($) + 3C($) → SiCl(S) + 2H(S) + Cl_Si_(S)
SiH_3_Cl + 2C($) → SiCl(S) + H(S) + H_2_	C_2_H_6_ + 2Si($) → 2C(S) + 3H_2_
C_2_H_4_ + 2Si($) → 2C(S) + 2H_2_	C_2_H_3_ + 2Si($) → C(S) + CH(S) + H_2_
C_2_H_2_ + 2Si($) → 2C(S) + H_2_	CH_4_ + Si($) → C(S) + 2H_2_
SiCl_3_ + 2C($) + Si($) → SiCl(S) + Cl_C_(S) + Cl_Si_(S)	SiCl_3_ + C($) + 2Si($) → SiCl(S) + 2Cl_Si_(S)
SiCl_3_ + 3C($) → SiCl(S) + 2Cl_C_(S)	SiHCl + C($) → Si(S) + HCl
SiCl + C($) → SiCl(S)	2Cl_C_(S) + SiCl_2_ → SiCl_4_ + 2C($)
SiCl_2_ + 2C($) → SiCl(S) + Cl_C_(S)	Cl_Si_(S) + Cl_C_(S) + SiCl_2_ → SiCl_4_ + Si($) + C($)
SiCl_2_ + Si($) + C($) → SiCl(S) + Cl_Si_(S)	

* In this table, S designates surface species, and Si($) and C($) represent Si and C surface sites, respectively. Subscripts C and Si indicate a molecule absorbed on C or Si sites.

**Table 3:** List of C or Si contained intermediate species participate as reactants.

C Contained Species	Si Contained Species
C_2_H_6_, C_2_H_4_, C_2_H_3_, C_2_H_3_, CH_4_	SiH_3_Cl, SiHCl_3_, SiH_2_Cl_2_, SiCl_4_, SiCl_3_, SiCl_2_, SiCl, SiHCl

## Data Availability

Not applicable.
